# Heterodimerization of β_2 _adrenergic receptor and somatostatin receptor 5: Implications in modulation of signaling pathway

**DOI:** 10.1186/1750-2187-6-9

**Published:** 2011-08-12

**Authors:** Rishi K Somvanshi, Nicole Chaudhari, Xiaofan Qiu, Ujendra Kumar

**Affiliations:** 1 Faculty of Pharmaceutical Sciences, The University of British Columbia, Vancouver, BC, Canada

**Keywords:** G-protein-coupled receptor, Human somatostatin receptor-5, β_2 _adrenergic receptors, Heterodimerization, Photobleaching-fluorescence resonance energy transfer and Somatostatin

## Abstract

**Background:**

In the present study, we describe heterodimerization between human-Somatostatin Receptor 5 (hSSTR5) and β_2_-Adrenergic Receptor (β_2_AR) and its impact on the receptor trafficking, coupling to adenylyl cyclase and signaling including mitogen activated protein kinases and calcineurin-NFAT pathways.

**Methods:**

We used co-immunoprecipitation, photobleaching- fluorescence resonance energy transfer and Fluorescence assisted cell sorting analysis to characterize heterodimerization between SSTR5 and β_2_AR.

**Results:**

Our results indicate that hSSTR5/β_2_AR exist as preformed heterodimers in the basal condition which is enhanced upon co-activation of both receptors. In contrast, the activation of individual receptors leads to the dissociation of heterodimers. Receptor coupling to adenylyl cyclase displayed predominant effect of β_2_AR, however, somatostatin mediated inhibition of cAMP was enhanced upon blocking β_2_AR. Our results indicate hSSTR5 mediated significant activation of ERK1/2 and inhibition of phospho-p38. The phospho-NFAT level was enhanced in cotransfected cells indicating the blockade of calcineurin mediated dephosphorylation of NFAT upon receptor heterodimerization.

**Conclusion:**

These data for the first time unveil a novel insight for the role of hSSTR5/β_2_AR in the modulation of signaling pathways which has not been addressed earlier.

## Background

We have recently described homo-and heterodimerization of somatostatin receptor (SSTR) subtypes and its functional consequences on receptor trafficking and signaling in response to agonist activation. SSTRs heterodimerization is not restricted to its own family but has also been demonstrated with other member of G-protein coupled receptors (GPCRs) family such as dopamine and opioid receptors as well as with the members of receptor tyrosine kinase family [1-4]. In several pathological conditions including neurodegenerative diseases and tumors of different origin, somatostatin (SST) via its five receptor subtypes plays crucial role and serves as an important therapeutic approach. Most recent example of clinical implication of heterodimerization is the development of chimeric molecules of hSSTR5 and dopamine receptor 2 in treatment of pituitary tumor [5,6].

Adrenergic receptors (ARs) specifically β_1_AR and β_2_AR are the prominent receptor subtypes from GPCR family and have provided first convincing evidence in support of GPCRs dimerization [7-13]. β_1_AR and β_2_AR exhibit some similarities, but also exert receptor specific role on signaling molecules including receptor dependent stimulation of apoptosis and mitogen activated protein kinases (MAPKs). Widespread distributions of AR subtypes in different tissues provide the broad spectrum of physiological importance specifically in cardiac physiology [10,14-17]. However, the direct mechanistic and physiological importance of the ARs in heart failure is derived from the β_1 _and β_2_AR knockout and the transgenic mice [16-19].

SSTR subtypes are also well expressed in cardiac tissues and have been attributed to the beneficial role in cardiac physiology and are associated with positive and negative contractile function in concentration dependent manner [20-22]. Most importantly in patients with pituitary tumor (acromegaly) and Huntington's disease (HD), the high mortality rate is associated with cardiovascular diseases [23]. These significant observations anticipate the possibility of functional interaction between SSTR and β-AR subtypes. SSTRs and β-ARs have been studied extensively for homo-and heterodimerization within the family and with dopamine and opioid receptors with physiological significance and clinical implications [2,4,8,13,24-30]. There is no direct evidence whether SSTR and β-AR subtypes functionally interact with each other. Although, as early as in 1985, a study has described that in rat brain astrocytes, SST enhanced the production of β-AR mediated cyclic adenosine monophosphate (cAMP) [31]. In addition, agonist occupied β-AR gets phosphorylated in presence of β-AR kinase and SST and isoproterenol displayed similar effect in promoting the translocation of β-AR kinase [32,33]. Recently, we have shown the distributional pattern and colocalization of SSTRs and β-ARs in H9c2 cells [34]. In addition, we have described the functional interaction between SSTR5 and β_1_AR in human embryonic kidney cells (HEK-293 cells) [35]. These studies further support our concept and are compelling evidences to predict the functional interaction between adrenergic and somatostatin receptors in a receptor specific manner.

Accordingly, in the present study by using morphological, biochemical and biophysical techniques, we studied the heterodimerization between G_i_-coupled hSSTR5 and G_s_-coupled β_2_AR in HEK-293 stably cotransfected with both receptors and compared with monotransfected cells. We also analyzed receptor trafficking, coupling to adenylyl cyclase and downstream signaling cascades including extracellular signal-regulated kinases (ERK1/2), p38, protein kinase A (PKA) and nuclear transcriptional factor (NFAT) in mono-and/or cotransfected cells. Our results showed that hSSTR5/β_2_AR exhibits heterodimerization in basal condition or upon combined activation and modulate signaling pathways in receptor specific manner.

## Materials and methods

### Materials

Somatostatin-14 was obtained from Bachem, Torrance, CA. β_2_AR agonist formoterol hemifumarate and antagonist ICI-118551 was purchased from Tocris Cookson Inc., Ellisville, Missouri, USA. The non-peptide agonist L-817818 (hSSTR5) was provided by Dr. S.P. Rohrer from Merck & Co [36]. Monoclonal and polyclonal antibodies against HA- and cMyc- and β-actin were procured from Sigma-Aldrich, Inc., St. Louis, MO. Fluorescein and rhodamine conjugated goat-anti-mouse and goat-anti-rabbit secondary antibodies were purchased from Jackson Immuno Research ON. Polyclonal antibodies for total and phospho-ERK1/2 (phosphorylation site-Thr202/Tyr204) and p38 (phosphorylation site- Thr180/Tyr182) were obtained from Cell Signaling Technology, Danvers, MA. Antibodies for total and phospho-PKA (phosphorylation site-Thr198) and NFAT (phosphorylation site-Ser265) were purchased from Santa Cruz Biotechnology, Santa Cruz, CA. cAMP assay kit was purchased from BioVision, Inc. CA, USA. Protein A/G-Agarose beads were procured from Calbiochem, EMD Biosciences, Darmstadt, Germany. Reagents for electrophoresis were purchased from BIO-RAD Laboratories Mississauga ON, Canada. 4',6-diamidino-2-phenylindole (DAPI) dihydrochloride was purchased from Molecular Probes, Inc., Eugene, OR. Reagents for cell culture were purchased from GIBCO, Invitrogen, Burlington, ON, Canada. Other reagents were of AR grade and were procured from various sources.

### Receptor Constructs and Cell Lines

cMyc-β_2_AR in pCDNA3.1^+^/Hygro vector (hygromycin resistance) was purchased from TOP Gene Technologies, Montreal, Canada. Construct of HA-SSTR5 was made using the pCDNA3.1^+^/Neo (neomycin resistance) as previously described [2,24,25,37]. The stable transfections of HA-hSSTR5 and cMyc- β_2_AR in HEK-293 cells were prepared by Lipofectamine transfection reagent as described [1,2,24,25,37]. Cotransfection of cMyc-β_2_AR in the HEK-293 cells stably expressing HA-hSSTR5 was performed using Lipofectamine transfection reagent and the cells were maintained in Dulbecco's MEM supplemented with 10% fetal bovine serum (FBS), 700 μg/ml neomycin and 400 μg/ml hygromycin as described earlier [1,2,24,25,37].

### Co-immunoprecipitation (Co-IP)

HEK-293 cells cotransfected with cMyc-β_2_AR/HA-hSSTR5 were treated with SST (1 μM) and formoterol (1 μM) alone or in combination for 30 min at 37°C. Membrane protein (250 μg) was solubilized in 1 ml of radioimmune precipitation assay buffer (RIPA Buffer, 150 mM NaCl, 50 mM Tris-HCL, 1% Nonidet P-40, 0.1% SDS, 0.5% sodium deoxycholate, pH 8.0) for 1 h at 4°C and Co-IP was performed as previously described [35]. Briefly, samples were incubated with 1 μg antibody overnight at 4°C. 25 μl of protein A/G-agarose beads were added to immunoprecipitate antibody for 2 h at 4°C. Beads were washed and solubilized in Laemmli sample buffer (Bio-Rad) and fractionated by electrophoresis on a 10% SDS-polyacrylamide gel. The fractionated proteins were transferred to a 0.2 μm nitrocellulose membrane and blotted with anti-HA or anti-cMyc antibody (dilution 1:500) for the expression of HA-hSSTR5 and cMyc-β_2_AR and detected by chemiluminescence using ECL Western blotting detection kit (Amersham) according to the manufacturer's instructions [24,37]. Images were captured using an Alpha Innotech FluorChem 8800 gel box imager (Alpha Innotech Co., San Leandro, CA).

### Photobleaching- Fluorescence Resonance Energy Transfer (Pb-FRET) Microscopic Analysis

HEK-293 cells expressing cMyc- β_2_AR/HA-hSSTR5 were grown on poly-D-lysine coated glass coverslips to 60-70% cell confluency. Cells were treated with 1 μM SST and 1 μM β_2 _agonist alone or in combination for 15 min at 37°C and fixed with 4% paraformaldehyde for 20 min on ice and were further processed for immunofluorescence immunocytochemistry [24,35,37]. Monoclonal anti-HA and polyclonal anti-cMyc primary antibodies were used followed by FITC- and Cy3- conjugated secondary antibodies to create donor-acceptor pair. The plasma membrane region was used to analyze the photobleaching decay on a pixel-by-pixel basis as previously described [24,37]. The FRET efficiency (E) was calculated in terms of a percent based upon the photo bleaching (Pb) time constants of the donor taken in the absence (D-A) and presence (D+A) of acceptor and relative FRET efficiency was calculated as previously described [35].

### Receptor Internalization

To study receptor internalization, HEK-293 cells stably expressing cMyc-β_2_AR/HA-hSSTR5 were grown on poly-D-Lysine coated coverslips to 60-70% cell confluency. Cells were treated with SST (1 μM) and formoterol (1 μM) alone or in combination for 15 min at 37°C and were processed for immunocytochemistry as previously described [25,35,37]. Receptor expression in both non-permeabilized and permeabilized cells was analyzed by using Leica DMLB microscope attached with the Retiga 2000R camera. DAPI dihydrochloride was used for nuclear staining. Adobe Photoshop was used to construct figure composites and merged images displaying colocalization were generated by using Image J software, NIH.

### Fluorescence assisted cell sorting (FACS) Analysis

The changes in cell surface expression of receptors and measurement of FRET was also performed by using a FACS. Approximately 2 × 10^6 ^cells were treated with SST, formoterol and CGP alone or in combination for 15 min at 37 C in DMEM. Cells were washed with FACS buffer (PBS pH7.4, 5% FBS, 2 mM EDTA), fixed in 4% paraformaldehyde and were processed for immunostaining. Anti-HA and anti-cMyc primary antibodies were used followed by FITC- and Cy3- fluorescence conjugated secondary antibodies. Non-stained cells were used to setup the background level of fluorescence whereas; control cells stained with either Cy3- or FITC- was used as fluorescence control. A BD LSRII flow cytometer, configured with a 488 nm and 561 nm laser was used for the experiments. The level of Cy3- was monitored using the 561 nm laser and a 610/20 emission filter to directly measure the β_2_AR expression levels. The cells were excited with the 488 nm laser first and the emission was detected using a 530/30 filter to detect the SSTR5 expression level whereas a 610/20 filter was used to detect any Cy3 emission due to FRET between SSTR5 and β_2_AR. Data analysis was done by using FlowJo 7.6 software.

### Receptor Coupling to Adenylyl Cyclase (AC)

Briefly, to determine the basal levels of cAMP, transfected cells were incubated for 30 min with receptor specific agonists alone or in combination at 37°C in presence of 0.5 mM 3-isobutyl-1-methylxanthine. Similarly, cells were also incubated for 30 min with receptor specific agonist alone or in combination at 37°C in presence of 20 μM forskolin (FSK) and 0.5 mM 3-isobutyl-1-methylxanthine. Control and treated cells were then scraped in 0.1 N HCl and cAMP was determined by immunoassay using a cAMP Kit from BioVision, Inc. CA, USA according to the manufacturer's guidelines [24,37].

To determine the G-proteins coupling with β_2_AR and SSTR5 in mono- and/or cotransfected cells, G-Protein antagonizing peptide (GPAP) or Melittin and Pertussis toxin (PTX) were used to inhibit G_s _and G_i _respectively. Mono-and cotransfected HEK-293 cells expressing cMyc-β_2_AR and/or HA-hSSTR5 were grown in 6 well culture plates and used at > 70% cell confluency for cAMP assay [37]. In addition to the receptor specific agonist/antagonists, cells were also treated with GPAP (5 μM) and Melittin (1 μM) for 2 h and PTX (100 ng/ml) for 16-18 h in DMEM at 37°C and processed for cAMP estimation.

### Western blot analysis

HEK-293 cells monotransfected with β_2_AR or hSSTR5 were treated with receptor specific agonist whereas cells coexpressing β_2_AR/hSSTR5 were treated with SST (1 μM), L-817818 (10 nM) and formoterol (1 μM) alone or in combination for 10 min and 30 min at 37°C. Whole cell lysate prepared from cells were fractionated via SDS-PAGE and transferred to a 0.2 μM nitrocellulose membrane. Immunoblotting for ERK1/2 and p38 were performed by using respective phospho-and total specific antibodies and the bands were quantified by densitometry using FluorChem software as described earlier [35]. β-actin was used as loading control.

To determine the expression of total- and phospho-PKA and NFAT, mono- and/or cotransfected HEK-293 cells were treated with 1 μM SST, 10 nM L-817818, 1 μM formoterol alone or in combination for 10 or 30 min at 37°C in the medium containing 1.8 mM or 2.5 mM Ca^2+^. Cells were further processed and western blot was performed accordingly as described earlier [24,37].

### Statistical Analysis

Results are presented as mean ± S.E unless otherwise stated. Statistical analysis was carried out using Graph Pad Prism 4.0 and statistical differences were taken at *p *values < 0.05. The results presented here represent three independent experiments.

## Results

### Human somatostatin receptor 5 and β_2 _adrenergic receptor are constitutive heterodimers

**Figure 1:**
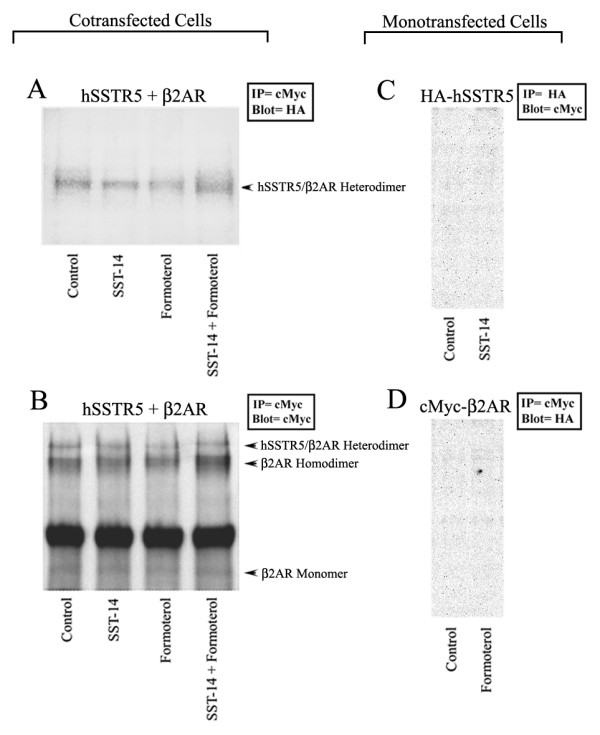
**Heterodimerization of hSSTR5 and β_2_AR in cotransfected HEK-293 cells**. Mono-and/or cotransfected cells were treated as indicated for 30 min at 37°C. A single band at the expected size of ~110 kDa an indicative of β_2_AR/hSSTR5 heterodimers was observed in β_2_AR immunoprecipitate **(arrow panel A)**. Same membrane was reprobed for β_2_AR expression using anti-cMyc specific antibody for the specificity of heterodimers **(arrow panel B)**. A band at the expected size of ~50 kDa was also seen for β_2_AR monomers. For specificity, monotransfected cells expressing SSTR5 or β_2_AR were used. No bands at the expected molecular weights were observed in either control or treated condition indicating the specificity of heterodimerization **(Panels C and D)**. Data are representative of three independent experiments.

To ascertain whether β_2_AR and hSSTR5 exists as heterodimers, we first determined heterodimerization using Co-IP in stably cotransfected HEK-293 cells. Membrane preparation from control and treated cells were immunoprecipitated for cMyc-β_2_AR and probed with antibody directed against HA to recognize HA-hSSTR5. As shown in Figure 1A, upon treatment with SST and formoterol alone or in combination, a band at ~110 kDa, the expected size of HA-hSSTR5/cMyc-β_2_AR heteromeric complex was detected in cMyc immunoprecipitate. To further confirm the specificity of the blot, same membrane was striped and reprobed with the anti-cMyc antibody to determine the expression of cMyc-β_2_AR. As shown in Figure 1B, monomers and homodimers of β_2_AR as well as heterodimers of hSSTR5/β_2_AR were observed at the expected molecular size of ~60 kDa, ~130 kDa and ~110 kDa respectively. The immunoprecipitate prepared from hSSTR5 or β_2_AR monotransfected cells probed reciprocally were devoid of any heteromeric complex (Figure 1C and 1D).

### Microscopic Photobleaching Fluorescence resonance energy transfer (Pb-FRET)

**Figure 2:**
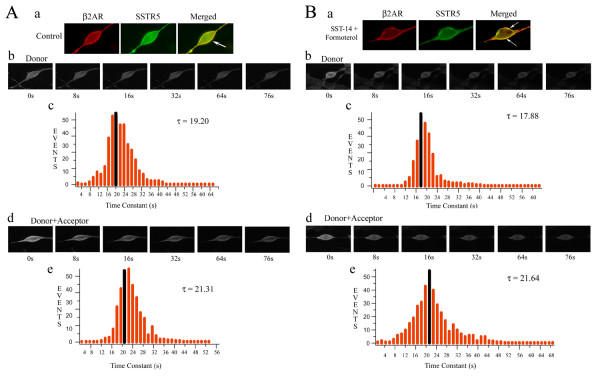
**Microscopic Pb-FRET analysis in HEK-293 cells coexpressing cMyc-β_2_AR/HA-hSSTR5**. **(A) **Representative photomicrographs of HEK-293 cells illustrating cMyc-β_2_AR (red), HA-hSSTR5 (green) and colocalization in merged image (yellow) **(Panel a)**. Pb-FRET microscopy on HEK-293 cells coexpressing cMyc-β_2_AR/HA-hSSTR5 was performed as described in Material and Methods. A selection of photomicrographs illustrating photobleaching profile of donor alone and in the presence of acceptor are shown in **panels b and d**. Histograms shown in **panels c and e **represents pixel by pixel analysis of time constant of donor and donor + acceptor and the mean time constant shown in black was calculated from a Gaussian distribution curve. Note the increased time constant of donor in presence of acceptor, indicating interactions between cMyc-β_2_AR/HA-hSSTR5. **(B) **HEK-293 cells expressing cMyc-β_2_AR/HA-hSSTR5 were treated with SST (1 μM) and formoterol (1 μM) in combination for 15 min at 37°C. Activation of hSSTR5 with SST and β_2_AR with formoterol simultaneously displayed increase in the effective FRET efficiency. Representative photomicrographs illustrating bleaching profile of the donor in the absence or presence of acceptor are shown in **panels b and d**. Histograms shown in **panels c and e **represent pixel by pixel analysis of time constant of the donor alone or donor + acceptor respectively in cells treated with SST and β_2_AR specific agonist together for 15 min. Data are representative of three independent experiments performed in duplicate. Approximately 60-70 cells were analyzed per experiment.

**Figure 3:**
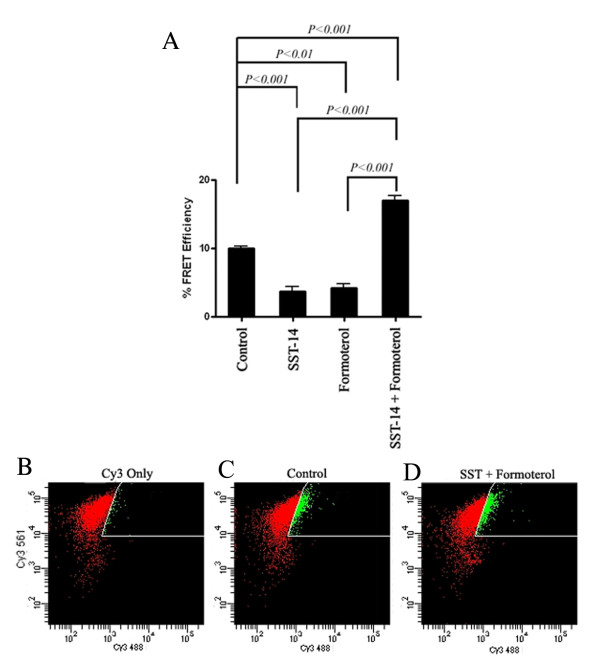
**Histogram and photomicrographs illustrating relative FRET efficiencies and FACS analysis in HEK-293 cells cotransfected with hSSTR5 and β_2_AR**. **(A) **Activation of hSSTR5 with SST or β_2_AR with formoterol displayed decrease in the FRET efficiency indicating the dissociation of receptor complex upon activation of individual receptors. In contrast, upon combined treatment with SST (1 μM) and formoterol (1 μM), cells displayed enhanced heterodimerization. Mean ± S.E. are representative of three independent experiments performed in duplicate. Data analysis was done by using ANOVA and *post hoc *Bonferroni's Multiple Comparison test to compare with control and treated conditions. **(B-D) **Approximately 2 × 10^6 ^cells were treated with receptor specific agonist as indicated in materials and methods section. Cells were washed with FACS buffer, fixed in 4% paraformaldehyde and were processed for immunostaining as described. BD LSRII flow cytometer, configured with a 488 nm and 561 nm laser was used for the experiments. Cy3 labeled cells were used as control **(Panel B)**. Note the increased emission in 610/20 channel when cells were excited with 488 nm laser in control **(Panel C) **as well as upon combined agonist treatment **(Panel D)**.

Heterodimerization between hSSTR5/β_2_AR as seen in Co-IP assay was further confirmed by microscopic Pb-FRET analysis (Figure 2A and 2B). Cells were processed for receptor expression by using monoclonal anti-HA and polyclonal anti-cMyc antibodies followed by FITC- (donor) and Cy3- (acceptor) conjugated secondary antibodies. Cells expressing HA-hSSTR5 and cMyc-β_2_AR displayed relative FRET efficiency of 10 ± 0.5%, indicating that hSSTR5/β_2_AR exists as preformed heterodimers at basal condition (Figure 2A and 3A). Interestingly, upon treatment with SST or formoterol alone, cells displayed 3.7 ± 1.3% and 4.2 ± 1.1% of relative FRET efficiency respectively in comparison to control. However, co-activation of hSSTR5 and β_2_AR exhibited 17.4 ± 1% of relative FRET efficiency, significantly higher than the basal level (Figure 2B and 3A). These data suggest that the simultaneous activation of both the receptors enhanced the formation of heteromeric complex between hSSTR5 and β_2_AR. Similar results were observed when directly labeled HA- and cMyc- antibodies were used in FRET analysis to support that FRET signals were not due to the aggregation of antibodies (data not shown).

### Fluorescence resonance energy transfer analysis by using FACS

To further confirm the results obtained from Co-IP and microscopic Pb-FRET analysis, non-invasive, sensitive and quantitative FACS analysis was employed to determine the receptor heterodimerization in cotransfected cells. Cells were processed as described in Materials and methods. To measure the FRET between SSTR5 and β_2_AR, Cy3- emission at 610/20 was detected upon excitation of FITC with 488 nm laser in double labeled cells (Figure 3B-D). As shown in the Figure 3B, no significant emission in 610/20 channel was detected when Cy3 labeled cells were excited with 488 nm laser (mean fluorescence intensity = 21.1). In contrast, when both the receptors were labeled with fluorescence conjugated antibodies, excitation with 488 laser in control resulted in the enhanced emission at 610/20 channel displaying mean fluorescence of 26.2, indicating FRET (Figure 3C). Upon treatment with receptors specific agonist alone, cells displayed mean fluorescence comparable to Cy3 labeled cells (~22.0). Interestingly, upon combined agonist treatment, increased FRET was observed with enhanced mean fluorescence of 28.8 indicating fostered heterodimerization (Figure 3D). Taken in consideration, these data indicate that heterodimerization observed between SSTR5 and β_2_AR by using Co-IP, Pb-FRET and FACS analysis is receptor specific.

### Receptor and agonist dependent internalization of hSSTR5 and β_2_AR using indirect immunofluorescence and FACS analysis

**Figure 4:**
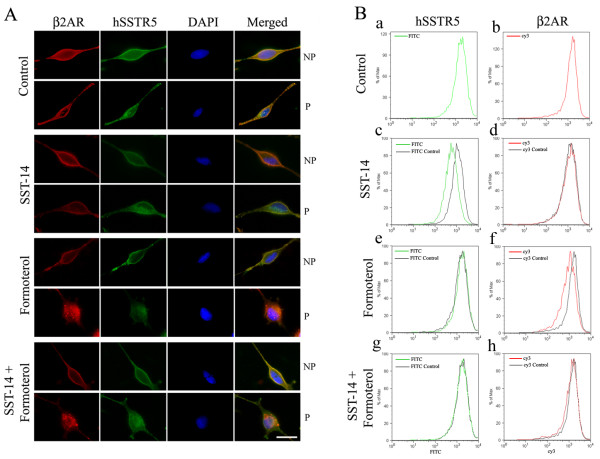
**Representative photomicrographs and FACS analysis illustrating receptor and agonist dependent internalization**. (**A) **The cells displayed colocalization at the membrane in non-permeabilized and intracellularly in permeabilized cells with distinct populations of hSSTR5 (green) and β_2_AR (red). Following treatment with SST, β_2_AR remain unchanged while hSSTR5 displayed agonist induced internalization. Upon activation with formoterol, β_2_AR internalized as indicated by the loss of β_2_AR and colocalization with hSSTR5 at cell surface which is accompanied with the increased intracellular expression. Combined treatment with SST and formoterol displayed colocalization between hSSTR5 and β_2_AR. In all representative photographs DAPI in blue color indicates nuclear staining. Scale bar = 10 μm. **(B) **BD LSRII flow cytometer configured with a 488 nm and 561 nm laser was used for the experiments. Cy3- (β_2_AR expression, **panels b, d, f and h**) was monitored using the 561 nm laser and a 610/20 emission filter whereas 488 nm laser was used to detect SSTR5 expression (FITC, **panels a, c, e and g**) and the emission was detected using a 530/30 filter. Cells were processed as described in materials and methods section. Note the left side shifts in the FITC and Cy3 expression **(Panels c and f) **indicating agonist dependent receptor specific internalization. No significant change in the expression pattern for the receptors was observed upon combined activation with both the ligands **(Panels g and h)**.

Previous studies have shown that hSSTR5 and β_2_AR displayed internalization upon agonist treatment [28,38]. Here, we determined the expression pattern of hSSTR5 and β_2_AR at the cell surface and intracellularly in cotransfected cells following treatment with the receptor specific agonists by using indirect immunofluorescence microscopy and FACS analysis. In control cells, hSSTR5 and β_2_AR exhibited strong colocalization at the cell surface than intracellularly (Figure 4A). Three different receptor populations were detected intracellularly i.e., either expressing hSSTR5 or β_2_AR and colocalization. hSSTR5 membrane expression was decreased upon treatment with SST and resulted in the loss of receptor colocalization at the cell surface. Increased expression of hSSTR5 was observed intracellularly without any significant effect on β_2_AR membrane expression (Figure 4A). In contrast, upon treatment with formoterol, β_2_AR internalized and resulted with the loss of receptor expression at cell surface. This resulted in the loss of colocalization with SSTR5 and increased intracellular expression of β_2_AR (Figure 4A). Importantly, simultaneous activation of both receptors displayed no significant changes in the membrane or intracellular expression as well as in colocalization when compared to the control.

Microscopic immunofluorescence internalization was further confirmed by using FACS analysis (Figure 4B, a-h). In control cells, both the receptors were well expressed on the membrane (Figure 4B, a and 4b). As shown in **panel c**, a significant decrease in the SSTR5 expression (FITC intensity) upon treatment with SST was observed, without any change in β_2_AR expression **(d)**. Upon treatment with formoterol, β_2_AR expression (Cy3 intensity) was reduced **(f)**, whereas, SSTR5 expression remained unaffected **(e)**. In combined treatment with both the agonist, no significant change in the emission at 530/30 and 610/20 filters was observed in comparison to control (Figure 4B, g and 4h). Taken together, results from immunofluorescence and FACS analysis indicate that agonist induced internalization of β_2_AR and SSTR5 is receptor specific and is independent of each other.

### Inactivation of β_2 _AR is required for the inhibition of cAMP via hSSTR5

**Figure 5:**
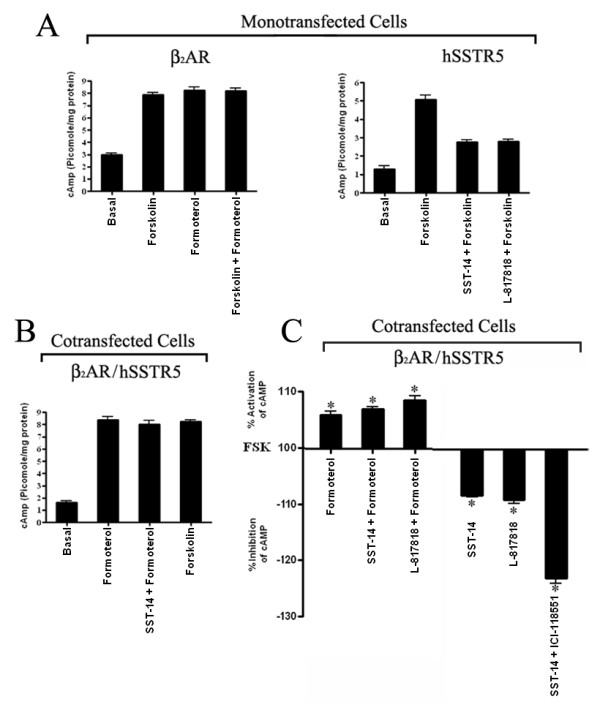
**Inactivation of β_2_AR enhanced hSSTR5 coupling to adenylyl cyclase**. Mono-and/or cotransfected HEK-293 cells were processed as described in materials and methods. In basal condition, cAMP level was relatively higher in cells expressing β_2_AR **(Panel A) **in comparison to hSSTR5 expressing cells **(Panel A)**. However, both the cell lines displayed increased cAMP in the presence of FSK. As shown, SSTR5 transfected cells exhibited the inhibition of cAMP in presence of SST and SSTR5 specific agonist. In cotransfected cells, basal level of cAMP was comparable to hSSTR5 expressing cells **(Panel B)**. Note the significant decrease in cAMP with SST in presence of β_2_AR antagonist **(Panel C)**. Mean ± S.E. are representative of three independent experiments performed in triplicate. Data analysis was done by using ANOVA and *post hoc *Dunnett's to compare against basal level (*, *p *< 0.01).

To better understand the molecular mechanism of this interaction, we first determined the changes in receptor coupling to AC in mono and/or cotransfected cells. In basal condition, cells transfected with β_2_AR showed relatively higher cAMP formation (2.99 ± 0.2 picomole/mg protein) in comparison to the cells transfected with hSSTR5 (1.3 ± 0.3 picomole/mg protein) (Figure 5A). In the presence of FSK, cells expressing β_2_AR or hSSTR5 displayed 7.9 ± 0.3 and 5.07 ± 0.41 picomole/mg protein of cAMP respectively. Monotransfected cells expressing hSSTR5 displayed inhibition of FSK stimulated cAMP levels by 55 ± 3% and 54 ± 3% upon treatment with SST and hSSTR5 specific agonist (L-817818) respectively (Figure 5A). In contrast, level of cAMP increased by ~3 folds (8.23 ± 0.5 picomole/mg protein) in β_2_AR monotransfected cells upon formoterol treatment. In cotransfected cells, the basal intracellular cAMP level (1.65 ± 0.2 picomole/mg protein) was elevated by ~4 folds in the presence of formoterol (8.4 ± 0.4 picomole/mg protein) (Figure 5B). Upon treatment with SST or L-817818, FSK stimulated cAMP was inhibited by 8.6 ± 0.3% and 9 ± 1.5% respectively. Conversely, following treatment with formoterol, FSK stimulated cAMP was increased by 6 ± 1%. Upon combined treatment with SST or L-817818 and formoterol cells displayed 7 ± 0.5% and 8.6 ± 1% increased cAMP in the presence of FSK. Most importantly, blockade of β_2_AR with receptor specific antagonist ICI-118551 (5 nM) in presence of SST resulted in enhanced inhibition of FSK stimulated cAMP levels by 23 ± 1.6% (Figure 5C). These data suggest that blockade of β_2_AR is essential for the inhibitory role of SST in cotransfected cells.

### Identification of G-Proteins involved in receptor coupling

**Figure 6:**
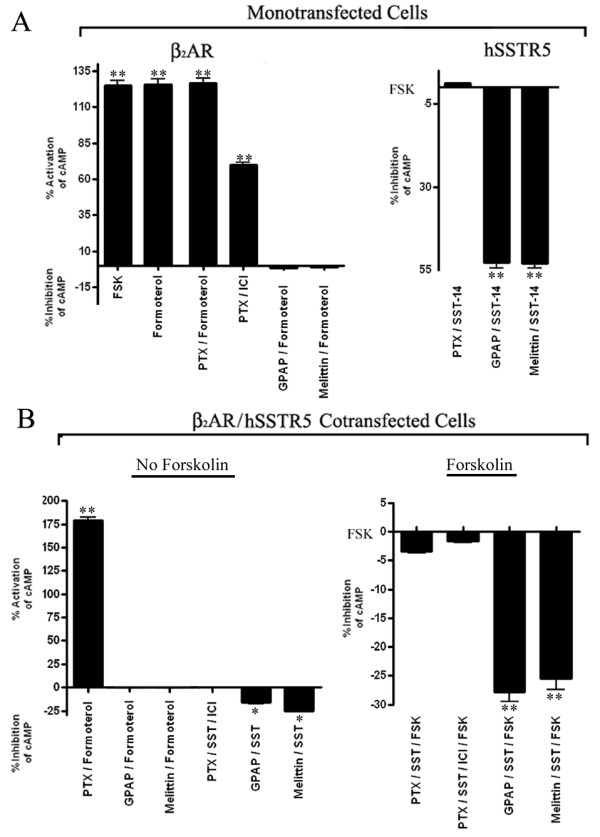
**Specificity of G protein coupling**. Mono-and cotransfected cells were treated with G_s _inhibitors GPAP (5 μM) and Melittin (1 μM) for 2 h; 100 ng/ml PTX (G_i _inhibitor) for 16-18 h in DMEM at 37°C and processed for cAMP estimation. GPAP and Melittin resulted in complete loss of formoterol induced cAMP levels, whereas PTX had no significant effect on increased cAMP levels upon formoterol treatment in mono and/or cotransfected cells **(Panels A and B)**. In contrast regulation of cAMP levels by SSTR5 were completely abrogated upon PTX treatment in mono or cotransfected cells. Mean ± S.E. are representative of three independent experiments performed in triplicate. Data analysis was done by using ANOVA and *post hoc *Dunnett's to compare against basal level (*, *p *< 0.05; **, *p *< 0.01).

GPCR coupling to AC is regulated by different G proteins including G_s_, G_i_, G_q _and G_12 _that play determinant role on intracellular cAMP levels. β-ARs typically interact with G_s _whereas SSTRs have been shown to couple G_i_. Excellent observations from Lefkowitz's laboratory and others have described that β_1_AR/β_2_AR also couple to G_i _and activate signaling pathways in distinct manner than G_s_[17,39]. To ascertain the specificity of G proteins involved in β_2_AR and SSTR5 mediated regulation of cAMP, we used G_s _inhibitors GPAP and Melittin and G_i _inhibitor PTX [40-42]. Mono and/or cotransfected cells were treated with GPAP, Melittin and PTX as described in methods section. As shown in Figure 6A, cells expressing β_2_AR displayed increased levels of cAMP by ~125% in presence of FSK or formoterol, the effect which was completely abrogated upon treatment with GPAP and Melittin. β_2_AR stimulated cAMP was not affected in presence of PTX in monotransfected cells (Figure 6A). SST mediated effect on cAMP inhibition was completely abrogated upon PTX treatment in SSTR5 monotransfected cells, whereas, GPAP and Melittin had no significant effect on cAMP levels (Figure 6A). These data in concurrence with previous studies indicate that β_2_AR and SSTR5 are coupled to G_s _and G_i _respectively [17,43]. In cotransfected cells, increased level of cAMP upon treatment with formoterol was abolished in presence of G_s _inhibitors GPAP and Melittin but not with PTX whereas, SST mediated inhibition of cAMP in presence of β_2_AR antagonist ICI was blocked with PTX (Figure 6B). Interestingly, SST displayed inhibition of cAMP by 25% and 27% in the presence of GPAP and Melittin respectively. These observations indicate that β_2_AR is predominantly coupled to G_s _in monotransfected as well as in cotransfected cells whereas, SSTR5 mediated regulation of cAMP is mediated by G_i _protein.

### Differential regulation of Protein kinase A by hSSTR5 and β_2_AR

**Figure 7:**
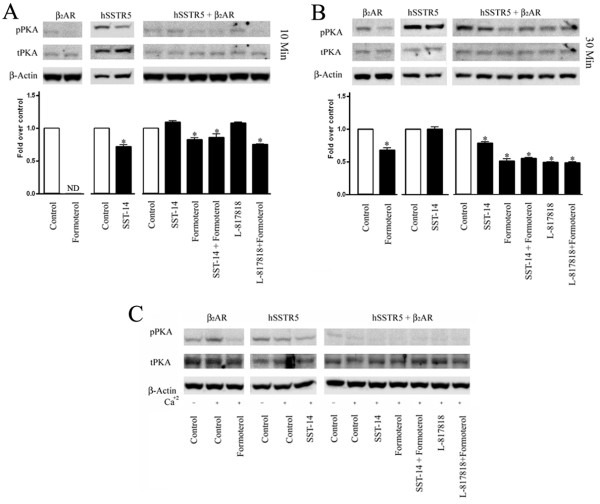
**Time and receptor specific changes in PKA phosphorylation**. To determine the expression levels of total and phospho-PKA, mono-and cotransfected cells were treated with SST, L-817818 and formoterol alone or in combination for 10 and 30 min at 37°C in the presence of basal **(Panels A and B) **and higher Ca^2+^**(Panel C)**. Upon 10 min treatment, cells expressing hSSTR5 displayed inhibition of PKA phosphorylation in the presence of SST whereas no significant phosphorylation of PKA was detected in cells expressing β_2_AR. In cotransfected cells, β_2_AR effect was predominant showing inhibition of pPKA with all treatments as indicated **(Panel A)**. Interestingly, PKA phosphorylation was enhanced in SSTR5 monotransfectant in control or in presence of SST for 30 min. With high Ca^2+ ^in culture medium, pPKA expression was higher in the presence of β_2_AR in comparison to hSSTR5 and was inhibited upon activation of receptors respectively. In cotransfected cells, enhanced PKA phosphorylation seen in monotransfected cells was completely diminished following agonist treatment **(Panel C)**. Densitometric analysis of western blot for pPKA is shown. Data analysis was done by using ANOVA and *post hoc *Dunnett's to compare against basal level (*, *p *< 0.01).

Previous studies have suggested the critical role of PKA in switching of G protein coupling of β_2_AR [44]. PKA upon activation phosphorylates β_2_AR at specific C-terminal domains which in turn promotes switching of receptor coupling from G_s _to G_i_, thus activating different signaling cascade [44]. To activate cAMP/PKA pathway mono-and/or cotransfected cells were grown in presence of basal Ca^2+ ^(1.8 mM) as well as in high concentration of Ca^2+ ^(2.5 mM) and cells were processed to determine the status of PKA phosphorylation. As shown in Figure 7A and 7B, β_2_AR or hSSTR5 monotransfected cells displayed receptor and agonist specific changes on PKA phosphorylation in presence of basal Ca^2+^. In β_2_AR monotransfected cells, formoterol decreased PKA phosphorylation upon 10 and 30 min treatment in comparison to control. In contrast, cells expressing hSSTR5 exhibited significantly decreased PKA phosphorylation in presence of SST at 10 min, whereas upon 30 min treatment phospho-PKA was comparable to control. In cotransfected cells, PKA phosphorylation was decreased upon 10 min treatment with formoterol alone or in combination with SST or SSTR5 agonist in comparison to control. However, following treatment for 30 min, the level of phospho-PKA was decreased significantly in comparison to control (Figure 7B). These results suggest that in cotransfected cells, β_2_AR mediated inhibition of phospho-PKA was predominant.

In comparison, at high Ca^2+ ^concentration in culture medium, the level of phospho-PKA increased in cells expressing β_2_AR and decreased in hSSTR5 transfected cells when compared to basal Ca^2+ ^concentration. SST or formoterol caused decrease in PKA phosphorylation in monotransfected cells (Figure 7C). However, cotransfected cells displayed complete inhibition of phospho-PKA upon treatment with SST, L-817818 (SSTR5 agonist) and formoterol alone or in combination. The effect observed in presence of high Ca^2+ ^indicates the blockade of cAMP/PKA mediated pathway with predominant role of β_2_AR.

### Receptor specific dependency in regulation of ERK1/2 phosphorylation

**Figure 8:**
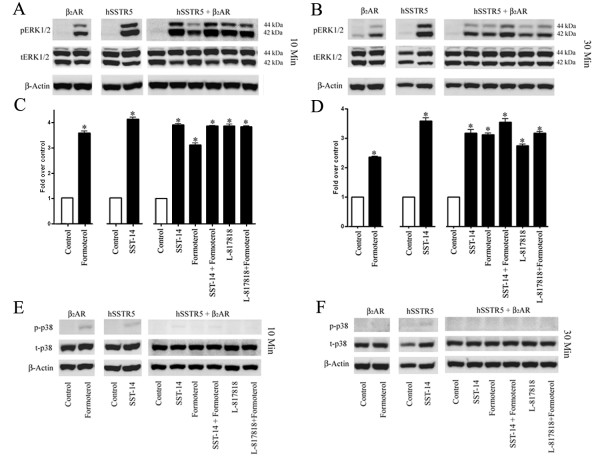
**Receptor mediated changes in ERK1/2 and p38 phosphorylation**. Mono-and cotransfected HEK-293 cells were treated with SST (1 μM), L-817818 (10 nM) and formoterol (1 μM) alone or in combination at 37°C for 10 and 30 min **(Panels A and B)**. In monotransfected cells, increased expression of pERK1/2 was observed with more significant changes in SSTR5 expressing cells. Similar pattern was also seen following 30 min incubation albeit to the lesser degree. In comparison, cotransfected cells displayed discernable changes at 10 or 30 min treatments **(Panels A and B)**. Densitometric analysis of western blot is shown for ERK1/2 **(Panels C and D)**. Data analysis was done by using ANOVA and *post hoc *Dunnett's to compare against basal level (*, *p *< 0.01). As shown in **panels E and F**, no significant expression of phosphorylated p38 was observed in mono-and/or cotransfected HEK-293 cells with or without treatment. Data are representative of three independent experiments.

The MAPK represents a critical signal transduction cascade involved in multitude of cellular processes and upon distinctive activation plays an important role in responding to the growth and stress stimuli [45]. To ascertain whether receptor heterodimerization and the changes in cAMP/PKA regulate downstream signaling cascades, we next examined the status of key MAPKs i.e., ERK1/2 and p38 in mono-and cotransfected HEK-293 cells. As shown in Figure 8A, the activation of β_2_AR and hSSTR5 enhanced phosphorylation of ERK1/2 in monotransfected cells. This effect was relatively more pronounced in SSTR5 transfected cells following treatment with SST for 10 and 30 min (Figure 8A and 8B). In comparison to monotransfectant, the cells coexpressing both the receptors, ERK1/2 phosphorylation was receptor and time dependent with the prominent effect of SST or SSTR5 agonist in combination with formoterol upon 10 min treatment (Figure 8A and 8B). In contrast, upon 30 min treatment with SST, L-817818 and formoterol alone or in combination, cells displayed decreased expression of phospho-ERK1/2 in comparison to 10 min treatment (Figure 8B). These results strongly support the G_i _mediated activation of phospho-ERK1/2 at early time point (10 min) whereas upon treatment for 30 min phospho-ERK1/2 is predominantly regulated by β_2_AR in heteromeric complex. Densitometric analysis was performed to quantify changes in phospho-ERK1/2 expression is presented in Figure 8C and 8D.

### Changes in p38 expression in cells expressing hSSTR5 and β_2_AR

Several previous studies have described that changes in the p38 expression are associated with pro and/or anti-apoptotic effects in receptor and tissue specific manner [45]. β_2_AR coupling to G_i _has been appreciated for its role in cell survival pathway due to anti-apoptotic action other than G_s _mediated signaling. In contrast, hSSTR5 is shown to exert antiproliferative effects. Whether or not β_2_AR encounter or potentiate this effect of hSSTR5 in cotransfected cells is not known. Here, we examined the status of phospho-p38 in mono-and cotransfected cells expressing β_2_AR and hSSTR5. Monotransfected cells displayed weak expression of phospho-p38 upon receptor specific activation at 10 min in comparison to control (Figure 8E). Following 30 min incubation in presence of receptor specific agonists phosphorylated p38 was not detected despite the expression of total p38 as indicated (Figure 8F). The phospho-p38 was not detected in cotransfected cells with or without receptor specific activation alone or in combination following 10 or 30 min treatments, whereas expression level of total p38 remained comparable in all the conditions as indicated in Figure 8E and 8F.

### The presence of β_2_AR and hSSTR5 regulates dephosphorylation and nuclear translocation of NFAT

**Figure 9:**
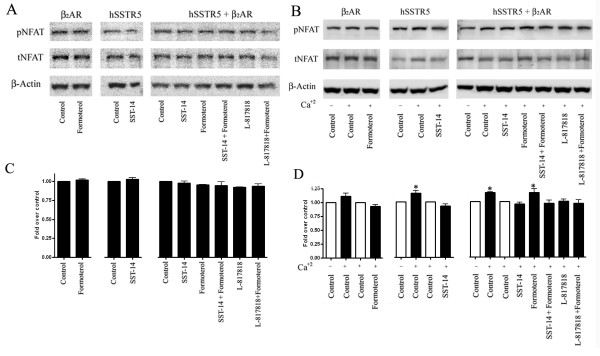
**hSSTR5 and β_2_AR blocks the dephosphorylation of NFAT**. Mono-and cotransfected HEK-293 cells were treated with SST (1 μM), L-817818 (10 nM), β_2 _agonist (1 μM) alone or in combination at 37°C for 30 min and analyzed for total and phosphorylated NFAT in presence of basal and elevated Ca^2+^. In the presence of basal Ca^2+ ^no significant changes were seen in NFAT phosphorylation in mono-and/or cotransfected cells expressing β_2_AR or hSSTR5 **(Panel A)**. In the presence of high Ca^2+ ^in culture medium, increased level of phosphorylated NFAT was observed in monotransfected cells when compared with basal Ca^2+ ^control. Cotransfected cells displayed increased phospho-NFAT upon formoterol treatment whereas NFAT phosphorylation remained comparable to control upon activation of SSTR5 alone or in combination with β_2_AR agonist **(Panel B)**. Densitometric analysis of western blot for pNFAT is shown in **panels C and D**. Data analysis was done by using ANOVA and *post hoc *Dunnett's to compare against control (*, *p *< 0.05).

Calcium mediated activation of Calcineurin induces dephosphorylation of NFAT transcription factor in the cytoplasm and increased dephosphorylation persuade its nuclear translocation which is associated with activation of several gene expression [46]. Accordingly, in this experiment we compared the expression of phospho-NFAT in mono-and/or cotransfected HEK-293 cells. As shown in Figure 9A, no significant changes in the expression of pNFAT were observed when β_2_AR or hSSTR5 monotransfected cells were treated with receptor specific agonists in presence of basal Ca^2+^. Comparable expression of phospho-NFAT was observed in β_2_AR and hSSTR5 cotransfected cells in control as well as treated conditions as indicated. However, the status of NFAT phosphorylation was elevated in HEK-293 cells expressing hSSTR5 or β_2_AR in presence of high Ca^2+ ^with no significant effect of receptor specific activation (Figure 9B). In cotransfected cells, Formoterol treatment enhanced the phosphorylation of NFAT significantly whereas SST or L-817818 alone or in combination with Formoterol the expression of phospho-NFAT was comparable to control. To quantify the changes in phospho-NFAT expression, densitometric analysis was performed (Figure 9C and 9D). These results provide direct evidence that β_2_AR and hSSTR5 ablated calcineurin mediated dephosphorylation and nuclear translocation of NFAT.

## Discussion

In the present study, we describe the heterodimerization between β_2_AR and hSSTR5 in stably cotransfected HEK-293 cells and characterized its role on key signaling pathways. Our study showed that β_2_AR and hSSTR5 exist as preformed heterodimers in basal condition and regulate receptor trafficking, coupling to AC and modulate the signaling cascades in receptor and time dependent manner. Although, β_2_AR has been studied extensively as a model of receptor dimerization, present study revealed for the first time that β_2_AR and hSSTR5 can function synergistically on selective MAPKs and cAMP dependent protein kinase A. Significantly, the effects of SSTR5 in association with β_2_AR are completely different from the hSSTR5/β_1_AR heterodimers as described recently [35].

The heterodimerization between chemokine receptor 4 and opioid receptor is stabilized in the presence of ligands for both protomers whereas, the activation of individual receptor prompted dissociation of heterodimeric complex and leads to the inactivation of signaling pathway [47]. In agreement with these observations by using conventional co-immunoprecipitation, microscopic Pb-FRET and FACS analysis, here we describe that β_2_AR/hSSTR5 exists as heterodimers in basal condition and heterodimerization was disrupted upon activation of β_2_AR or hSSTR5 alone. Devi et al., described that simultaneous activation of opioid receptor and β_2_AR enhanced the heterodimerization [27]. Consistent with these observations, we here describe that synergistic activation of β_2_AR and hSSTR5 resulted in enhanced heterodimerization while activation of individual receptor was devoid of such effect [27]. Interestingly, this is just opposite to our recent study demonstrating that co-activation of hSSTR2/hSSTR5 is not required for heterodimerization and the activation of single protomer is fully capable to exhibit the formation of heterooligomers [24]. Increased FRET efficiency upon activation of both receptors suggests the changes in conformation and orientation of receptor distribution at cell surface. In contrast, the activation of single receptor prompted the dissociation and resulted in loss of FRET efficiency. Consistent with these observations, we have recently shown the activation of individual receptor in cells cotransfected with β_1_AR/hSSTR5 promotes the formation of homodimers preferentially over heterodimerization of the receptors [35].

FACS is emerging as one of the most modern and reliable technique for determining fluorescence resonance energy transfer along with quantification of cell surface expression of receptors. FACS is highly sensitive and large number of cells live or fixed can be analyzed in a short duration of time [48]. We took an advantage of the versatility of FACS, and determined the FRET between β_2_AR and hSSTR5 upon treatment with receptor specific agonist alone or in combination. The FACS-FRET is quantitative, highly accurate, reproducible method and the results obtained further supports the data procured from the classical method like Co-IP or the state of art biophysical method Photobleaching-FRET analysis.

Receptor heterodimerization at the cell surface serves as regulatory mechanism for receptor expression and receptor specific internalization in presence of specific ligand [49-51]. As shown earlier, β_2_AR exhibits internalization in agonist dependent manner whereas β_1_AR is confined at the cell surface and impairs β_2_AR internalization in β_1_AR/β_2_AR cotransfected cells [28]. SSTR subtypes upon ligand induced activation displayed receptor specific internalization in time and temperature dependent manner except hSSTR1 that is rather upregulated at cell surface and only internalize with hSSTR5 in heteromeric complex [38]. We have recently shown that β_1_AR regulates internalization of hSSTR5 which displays colocalization at the cell surface [35]. Here, we described that cells cotransfected with β_2_AR and hSSTR5 exhibit β_2_AR or SSTR5 internalization upon receptor specific activation. Most importantly, synergistic activation of both receptors exhibit strong colocalization at cell surface and may account for enhanced FRET efficiency as discussed above. Our results are consistent with previous study describing the receptor specific internalization and trafficking of β_2_AR and δ or κ opioid receptors in heterodimeric complex [27].

All five human SSTR subtypes inhibit adenylyl cyclase-cAMP via pertussis toxin-sensitive G_i _proteins [37]. In contrast, ARs are positive regulators of AC via coupling to G_s _[44]. However, β_1_AR and β_2_AR upon heterodimerization display no significant changes in cAMP levels in comparison to monotransfected cells [28]. Interestingly, β_2_AR can also couple to G_i _and inhibit AC activity [52]. In addition, selective activation of β_2_AR in the cells coexpressing OR/β_2_AR increased cAMP whereas, activation of ORs resulted in the inhibition of cAMP [27]. Our results strongly support the notion that presence of both receptor subtypes exerts opposing effect in regulation of cAMP with prominent role of β_2_AR upon combined treatment. Most importantly, blockade of β_2_AR in the presence of β_2_AR antagonist enhanced the inhibitory role of SST on FSK stimulated cAMP. Our observations implicate that inactivation of β_2_AR is the prerequisite to unmask the SST mediated inhibitory role in regulation of cAMP levels in cells cotransfected with β_2_AR and SSTR5. These results are consistent with our previous study describing the effect of β_1_AR and SSTR5 on cAMP regulation although the inhibitory effect of SST was more pronounced in β_1_AR/SSTR5 transfected cells [35].

The activation of cAMP/PKA, MAPK and calcineurin dependent NFAT dephosphorylation and nuclear translocation of NFAT are intimately associated events in regulation of adrenergic receptor functions. PKA expression is suppressed upon activation of SSTRs suggesting that cAMP/PKA is inhibited due to the activation of G_i_. Although, β_2_AR mediated activation of cAMP/PKA is well established via coupling to G_s _however, the role of G proteins coupling in cotransfected cells has not been studied in detail. The results described here indicate that heterodimerization may not involve in promoting switching of G protein Coupling. Further in addition, previous studies have shown that β_2_AR coupling to dual G proteins shifts towards G_i _upon PKA mediated phosphorylation of β_2_ARs [44]. This transition from G_s _to G_i _is most controversial and poorly understood and further studies are required to resolve such discrepancies. Recently, using Fluorescence fluctuations of quantum-dot sensors report that β_2_AR agonist mediated PKA activation is completed within 3s [53]. Whether prolonged activation of β_2_AR involved in this transition is not known. Taken together, the time dependent changes described here suggest that activation of PKA at early time points might be sufficient enough to phosphorylate β_2_AR to involve G_i _mediated effect which may partially recover upon 30 min. Most importantly, in cotransfected cells, complete inhibition of PKA phosphorylation at high Ca^2+ ^further strengthens the concept of G_i _mediated coupling of both the receptor subtypes. These data strongly suggest the crucial role of hSSTR5/β_2_AR heterodimerization is attributed to G_i _mediated effect of β_2_AR. The results presented here are significantly distinct from the β_1_AR/SSTR5 transfected cells, as the activation of SSTR5 enhanced PKA phosphorylation and Isoproterenol treatment resulted in inhibition of PKA phosphorylation in cotransfected cells [35].

In addition to receptor coupling to cAMP and PKA phosphorylation, the changes in MAPKs including phosphorylation of ERK1/2 and p38/JNK pathways have been studied extensively as functional consequence of GPCR activation and dimerization. In contractile cells, G_i _mediated activation of ERK via GPCRs has been reported [54]. Consistent with these studies, β_2_AR internalization is associated with the inhibition of ERK phosphorylation [28]. Here, we describe the sustained activation of ERK1/2 in cells expressing hSSTR5 and β_2_AR upon synergistic activation of hSSTR5/β_2_AR. Such activation of ERK directly correlates with receptor heterodimerization. Whether or not this effect of SSTR5 is associated with anti-proliferative effect of SST via activation of ERK needs to be determined. Interestingly, the effect of β_1_AR or β_2_AR agonist on the phosphorylation of ERK1/2 has significantly diverse effects. As previously shown, Isoproterenol treatment resulted in increased phospho-ERK1/2 expression, whereas formoterol has inhibitory effect on phosphorylation of ERK1/2 [35].

Consistent with the changes in ERK1/2, the loss of p38 in cotransfected cells is governed with the prominent role of hSSTR5. Taken together increased ERK1/2 and loss in p-p38 expression might implicate in inhibition of cell proliferation. An antagonist of p38 (SB203580) reduce the agonist induced hypertrophy and reduced p38 signaling in the heart promote myocyte growth through a mechanism involving enhanced calcineurin-NFAT signaling [45]. Thus, calcineurin-NFAT and JNK signaling pathways crosstalk represents a critical mechanism that regulate cell physiology specifically in cells of cardiac origin [46]. HEK-293 cells express calcineurin endogenously and its inactivation inhibits NFAT dephosphorylation and translocation to nucleus, the process which is regulated by Ca^2+^[55-57]. In case of β_1_AR/hSSTR5 heterodimers, the dominant role of SSTR5 was evident as β_1_AR activation resulted in loss of pNFAT levels whereas SSTR5 displayed consistent expression of pNFAT [35]. We here describe that upon formation of β_2_AR/hSSTR5 heterodimers, NFAT dephosphorylation is blocked significantly and both the receptors contribute equally to exert this effect. Increased NFAT phosphorylation in cotransfected cells provides the first evidence that β_2_AR/hSSTR5 heterodimers blocks NFAT dephosphorylation and its nuclear translocation.

Although, SSTR5 heterodimerizes with β_1_AR as well as β_2_AR however, it exhibit distinct regulation of signaling pathways in both the cases. In cells transfected with β_1_AR/SSTR5, the role of SSTR5 in regulation of signaling is predominant whereas in cells transfected with β_2_AR/SSTR5, the effect of SSTR5 is synergistic. In conclusion, data presented here provides new insight for the role of hSSTR5 and β_2_AR which might have physiological significance and therapeutic implications in cardiac physiology in pituitary tumor and Huntington disease. The results presented in this study specifically changes in signaling pathways including cAMP, PKA, ERK1/2 and NFAT in hSSTR5/β_2_AR complex are quite interesting. Whether SSTRs and β-ARs colocalize in cardiac tissue *in vivo *is largely elusive and further studies are warranted prior to draw any conclusion for the role of SSTRs and β-ARs in this direction.

## Abbreviations

**AR: **adrenergic receptors; **cAMP: **cyclic adenosine monophosphate; **D and A: **donor and acceptor; **D-PBS: **Dulbecco's phosphate buffered saline; **ERKs: **extracellular signal-regulated kinases; **FSK: **forskolin; **GPAP: **G-Protein Antagonizing Peptide; **GPCRs: **G-protein coupled receptors; **hSSTR: **human somatostatin receptor; **HA: **hemagglutinin; **HEK-293: **human embryonic kidney-293; **JNK: **c-Jun N-terminal kinases; **MAPK: **mitogen-activated protein kinase; **NFAT: **Nuclear factor of activated T-cells; **Pb-FRET: **Photobleaching- fluorescence resonance energy transfer; **PKA: **Protein Kinase A; **PTX: **Pertussis Toxin; **SST: **somatostatin-14.

## Competing interests

The authors declare that they have no competing interests.

## Authors' contributions

UK. designed the experiments. RKS, NC and XQ performed the experiments. UK and RKS analyzed the results and wrote the manuscript. All authors read and approved the final version of the manuscript.
